# The Effect of Body Weight Support Treadmill Training on Gait Recovery, Proximal Lower Limb Motor Pattern, and Balance in Patients with Subacute Stroke

**DOI:** 10.1155/2015/175719

**Published:** 2015-11-16

**Authors:** Yu-Rong Mao, Wai Leung Lo, Qiang Lin, Le Li, Xiang Xiao, Preeti Raghavan, Dong-Feng Huang

**Affiliations:** ^1^Department of Rehabilitation Medicine, Guangdong Provincial Research Center for Rehabilitation Medicine and Translational Technology, The First Affiliated Hospital, Sun Yat-sen University, Guangzhou 510080, China; ^2^Motor Recovery Research Laboratory, Department of Rehabilitation Medicine, RUSK Rehabilitation, New York, NY 10016, USA

## Abstract

*Objective*. Gait performance is an indicator of mobility impairment after stroke. This study evaluated changes in balance, lower extremity motor function, and spatiotemporal gait parameters after receiving body weight supported treadmill training (BWSTT) and conventional overground walking training (CT) in patients with subacute stroke using 3D motion analysis. *Setting*. Inpatient department of rehabilitation medicine at a university-affiliated hospital. *Participants*. 24 subjects with unilateral hemiplegia in the subacute stage were randomized to the BWSTT (*n* = 12) and CT (*n* = 12) groups. Parameters were compared between the two groups. Data from twelve age matched healthy subjects were recorded as reference. *Interventions*. Patients received gait training with BWSTT or CT for an average of 30 minutes/day, 5 days/week, for 3 weeks. *Main Outcome Measures*. Balance was measured by the Brunel balance assessment. Lower extremity motor function was evaluated by the Fugl-Meyer assessment scale. Kinematic data were collected and analyzed using a gait capture system before and after the interventions. *Results*. Both groups improved on balance and lower extremity motor function measures (*P* < 0.05), with no significant difference between the two groups after intervention. However, kinematic data were significantly improved (*P* < 0.05) after BWSTT but not after CT. Maximum hip extension and flexion angles were significantly improved (*P* < 0.05) for the BWSTT group during the stance and swing phases compared to baseline. *Conclusion*. In subacute patients with stroke, BWSTT can lead to improved gait quality when compared with conventional gait training. Both methods can improve balance and motor function.

## 1. Introduction

The worldwide prevalence of stroke was estimated in 2010 to be 33 million. Stroke is the second-leading global cause of death behind heart disease [1]. The incidence and prevalence of stroke in China are highest in the world [2, 3]. According to a recent epidemiological study on the global burden of stroke, the incidence of stroke in China has increased from 226/100,000 person-years in 1990 to 240/100,000 person-years in 2010 [1]. Walking ability and ambulatory independence determine the level of disability and likelihood of institutionalization in patients with hemiplegia [4, 5]. The degree to which gait can be restored after stroke is related to both the initial impairment in walking ability and the severity of lower extremity paresis [6, 7]. Early intervention with physical therapy to restore walking after stroke was recommended to improve motor function and decrease disability [8, 9]. Previous kinematic data indicated better outcome for patients who started rehabilitation programme at early stage than those who started late [10].

Gait performance is an indicator of mobility impairment and disability after stroke [6]. It predicts mortality, morbidity, and risk of future stroke [5, 11, 12]. Gait speed is responsive to short-term rehabilitation [8]. An improvement in gait speed of 0.16 m/s can reduce the level of assistance in patients with subacute stroke and was recommended to be the minimum clinically significant difference [13]. The control of gait involves the planning and execution from multiple cortical areas, such as secondary and premotor cortex [14, 15]. Stroke patients often have gait impairment such as decreased gait speed and asymmetrical gait cycle [16] as a result of cortical reorganization [17, 18]. Repetitive mass motor task practice had been shown to facilitate neuroplasticity and brain reorganization in stroke patients, resulting in enhanced motor recovery after stroke [19–23]. Near-infrared spectroscopic imaging and functional magnetic resonance imaging have demonstrated treatment induced changes of brain activation pattern during walking and after gait training [18, 24, 25]. Abnormal gait pattern in stroke patients is characterized by altered kinematic and kinetic parameters of hip, knee, and ankle joint during a gait cycle. Published studies have shown that proximal lower limb control plays a key role in improving gait speed and walking performance after stroke [26, 27]. The greater hip sagittal range of motion could avert circumduction profiles [28].

Body weight supported treadmill training (BWSTT) is a task-oriented technique for gait restoration after stroke [29–31]. BWSTT has the advantage over conventional therapy as it offers higher intensity, more repetitive and task-oriented practice over the same period of time when compared to conventional therapy [32]. Several studies have showed that BWSTT was more effective in gait speed improvement than regular physiotherapy [29, 31–34]. It has been demonstrated that BWSTT induces changes in corticomotor excitability which lead to improved balance and gait performance with chronic stroke [18]. However, other studies have reported that BWSTT was not superior to conventional gait training [35, 36]. Recent studies have reported that BWSTT can increase walking endurance in the subacute stage after stroke, but no improvement was reported in balance and 10 m gait speed [37]. To date, there are very few studies that have used gait analysis to show how the improvements in gait parameters come about after BWSTT or conventional therapy. There is still a lack of basic understanding of gait training on human locomotion [38]. Since gait impairments are a result of deficient neuromuscular control, a better understanding of the impact of gait training on lower limb motor pattern is therefore essential. Improvement on individual biomechanical subtasks of walking such as leg swing or balance control is positively associated with walking performance [39]. The purpose of this study is to investigate the changes in spatiotemporal characteristics of gait after BWSTT intervention and conventional therapy (CT), using three-dimensional motion analysis of gait in patients with stroke at the subacute stage. Based on corticomotor excitability theory and the advantage of BWSTT over CT, it is hypothesized that BWSTT training is superior to CT training in improving kinetic and kinematic gait parameters from the period before to the one after training. In addition, we investigated the impact of BWSTT on balance and lower extremity impairment when compared to CT.

## 2. Methods

### 2.1. Subjects

**Figure 1:**
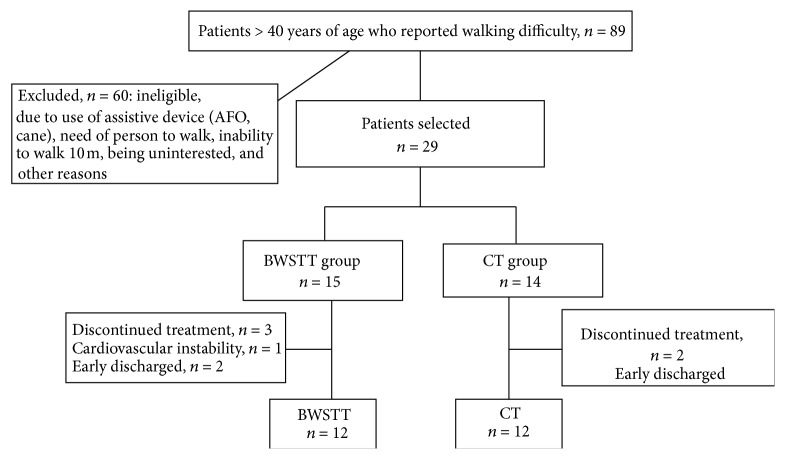
Flowchart for subjects selection process.

Subjects with subacute stroke (18 to 76 days after stroke) were recruited for this study and were randomly assigned to the BWSTT and CT groups (Figure 1). The inclusion criteria for the hemiparetic subjects were (1) stroke confirmed by computed tomography or MRI; (2) unilateral hemiparesis for no more than 3 months resulting from first stroke; (3) residual gait impairment, defined by an abnormal 10 m walk time according to age (age < 60 = 10 seconds or longer or 1 m/s; age 60 to 69: 12.5 seconds or longer or 0.8 m/s; age ≥ 70: 16.6 seconds or longer, <0.6 m/s) [24]; and adequate mental and physical capacity to attempt the tasks as instructed (Mini Mental State Examination score ≥ 27, average modified Ashworth scale score at hip, knee, and ankle ≤ 2).

Exclusion criteria were the presence of significant medical complications or unstable vital signs that precluded participation in the study. Twelve healthy adults matched for age, weight, leg length, and gender were also recruited to provide reference data for gait analysis. This study was approved by the Human Subjects Ethics Subcommittee of the First Affiliated Hospital, Sun Yat-sen University, China. Written consent was obtained from all participants prior to the experiment.

### 2.2. Training Protocol

All recruited stroke subjects received two hours of rehabilitation every weekday which was the standard of care in China. This included 60 minutes each of physical therapy and occupational therapy. The physical therapy sessions consisted of approximately 20–40 minutes of therapeutic exercises and 20–40 minutes of gait training. For gait training, subjects were randomly allocated to either BWSTT or conventional overground training for a period of 3 weeks. The therapeutic exercises component in all subjects remained the same and included range of motion and strengthening exercises, as well as facilitation techniques to recruit muscle activity on the paretic extremity. Occupational therapy session includes the use of functional stimulation and self-exercise program. All physical therapists involved in the study were trained on the protocol. Participants' daily compliance with the protocol was documented. In addition, the entire rehabilitation team was educated on the study protocol to ensure compliance when participants were not working with study-designated therapy staff.

The equipment for BWSTT consisted of a standard treadmill fitted with the weight supporting apparatus (Noramco Fitness and SpinoFlex, USA). The patient wore a modified mountain climber's harness with an adjustable belt around the pelvis and thigh and an adjustable belt above to support their body weight. A physiotherapist assisted with leg propulsion if the patient could not lift his/her paretic leg. At the beginning of training, some subjects needed two therapists to guide the movement of the pelvis forward and to flex and extend the hemiplegic leg during the swing and stance phases of gait. The initial body weight support was set at 30%~40%, the speed of the treadmill was set at 0.5 mph (miles per hour), and the duration of training was for 20 minutes. As treatment progressed, the body weight support was gradually decreased and the velocity was gradually increased. The two parameters were not changed simultaneously. By the third week the treadmill speed was increased to 2.5 mph and duration of training increased to 40 minutes. The training parameters were based on recommendation from previous literature [6]. Subjects were consulted throughout the training for fatigue level and tolerance of progression.

The CT group received individualized overground gait training by a physiotherapist for 30 minutes, five days a week, for three weeks. The training was based on the principles of neurodevelopmental therapy (Bobath method).

Heart rate and blood pressure were monitored in both groups before and after each session and during break using a digital sphygmomanometer.

### 2.3. Gait Analysis Protocol

Vicon Motion Analysis System (VICON MX13, VICON Peak, Oxford, UK) and two AMTI force plates (AMTI, OR6-7, Watertown, MA, USA) with sampling frequency of 1000 Hz recorded the joint angles and moments of the lower extremity in the sagittal plane simultaneously.

Six infrared 100 Hz cameras recorded the locations of 16 passive reflective markers taped to the skin overlying bony landmarks of the pelvis and both lower limbs, including the sacrum at the level of the posterior superior iliac spines, anterior superior iliac spines, lower lateral one-third and half-way points on the thighs, lateral epicondyle of knees, lower lateral one-third and half-way pints on the shank, lateral malleolus, the second metatarsal head, and the calcaneus at the same height as the second metatarsal head. The data were captured using Vicon Nexus (version 1.7.1) and Plugin Gait.

During gait analysis, all subjects were asked to walk back and forth at a self-selected walking speed on a 10-meter carpet. The subjects walked without canes, orthoses, or other assisted devices during the assessments. Five successful gait cycles (defined as one foot on one force plate) were selected for analysis at baseline and after 3 weeks of training. Marker trajectories were sampled at 100 Hz for the calculation of lower extremity joint angles and moments. Each walking trial was normalized to the total duration of the gait cycle (one foot strike to the next foot strike). Stance and swing phases of the gait cycle were presented as a percentage. The joint angles and moments were also normalized to the height and weight of each subject.

Lower limb impairment and balance were measured by Fugl-Meyer Lower Extremity Assessment (FMA-LE) [40] and the Brunel balance assessment [41, 42] scale. Measurements were recorded in stroke subjects at baseline and after 3 weeks of training by an examiner who was blinded to group assignment.

### 2.4. Data Processing

Spatiotemporal gait parameters, joint angles, and moment of the lower limb were processed using Polygon (version 3.5.1) and the mean data from 5 walking cycles were computed for each subject. The spatiotemporal parameters computed were cadence, stride time and length, step time and length, and walking speed. Kinematic and kinetic parameters of joint angles and moments at ankle, knee, and hip joints were recorded in the sagittal plane. Parameters were recorded at (1) maximum extension during the stance phase; (2) maximum flexion at the hip and knee joints during the swing phase; (3) plantarflexion during push-off; and (4) dorsiflexion during the swing phase of the gait cycle [43]. Spatiotemporal gait parameters of (1) cadence; (2) stride length; (3) stride time; (4) step length; (5) step time and gait speed; (6) peak angular flexion and extension; and (7) peak moments flexion and extension were recorded at the hip, knee, and angle joints in the sagittal plane.

### 2.5. Statistical Analysis

Data analyses were performed using SPSS version 15.0. Descriptive statistics were computed for demographic characteristics and for all parameters. Anthropometric data (age, body weight, postinjury date, and leg length) for the three groups were compared using one-way ANOVA at baseline. Paired samples *t*-test was used to assess the differences between pre- and postintervention within each group. Between-group differences were assessed by independent samples *t*-test. Statistical significant level was set as *P* < 0.05 (2-tailed).

## 3. Results

Twenty-nine stroke subjects who met the inclusion criteria were recruited. Five subjects were dropped out from the study. One subject experienced changes in health status unrelated to the study and four subjects were prematurely discharged from the hospital due to medical insurance issues. Twenty-four stroke subjects who completed all experimental protocols were included in the final data analysis. The two stroke groups showed no statistical significant differences in age and other anthropomorphic parameters. They were matched with age, weight, leg length, and gender to a healthy control group. There is no statistical significant difference in FMA-LE and Brunel balance tests between the two stroke groups at baseline (Table 1).

### 3.1. Balance and Lower Extremity Function

After three weeks of training, both BWSTT and CT groups improved significantly on the FMA-LE and on the Brunel balance scale (Table 2). The between-group differences were not statistically significant.

### 3.2. Spatiotemporal Parameters

The BWSTT group improved significantly in all spatiotemporal gait parameters during a gait cycle after interventions, whereas the CT group did not (Table 3). Cadence and gait speed were significantly higher in the BWSTT group than CT group after training.

### 3.3. Kinematic and Kinetic Parameters during Gait

**Figure 2:**
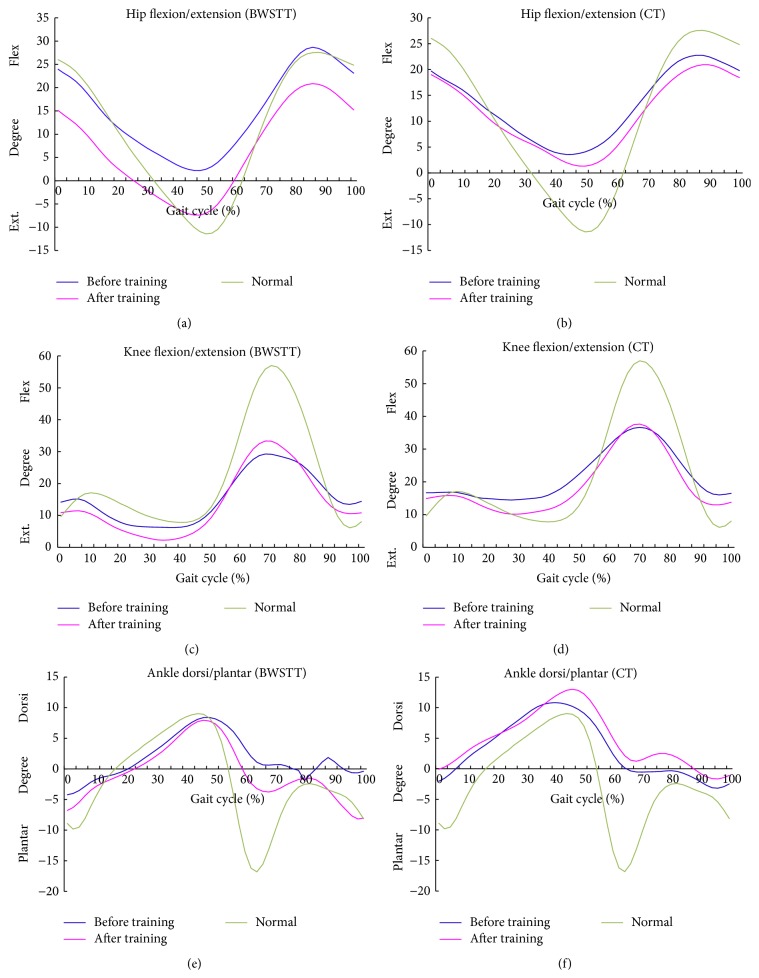
Average curves of lower limb kinematic at the sagittal plane for normal, BWSTT, and CT group. For the clearance of the comparison, the standard deviation was not shown in the figures.

Averaged kinematic trajectories of the hip, knee, and ankle joints in healthy controls and subjects with stroke in the BWSTT and CT groups are shown in Figure 2. Subjects with stroke were able to flex their hip joints comparable to the healthy control group but could not extend the hip adequately before training (Figures 2(a) and 2(b)). BWSTT group showed significantly reduced hip flexion and increased peak hip extension after training (Table 4), whereas the CT group did not. There were no significant differences in the angle of knee flexion or extension and ankle dorsiflexion or plantarflexion or in the peak moments at the hip, knee, or ankle joints in either the BWSTT group or the CT group (*P* > 0.05) after interventions (Table 4).

## 4. Discussion

This study sought to investigate the effects of BWSTT and CT on lower extremity motor functions, balance, spatiotemporal gait parameters, and kinetic and kinematic parameters during a gait cycle in subjects with subacute stroke. After BWSTT or overground gait training for three weeks, both groups demonstrated improvement of lower extremity motor function and balance capacity. The BWSTT training group demonstrated significant improvement in kinematic parameters of lower limb joints and gait patterns which was not observed in the CT group.

### 4.1. Balance and Lower Extremity Motor Functions

Previous studies on functional recovery in animal models [44] and patients with stroke [45–47] have demonstrated that early treatment and training can facilitate improvement in motor functions and balance. In previous studies that compared BWSTT with conventional overground gait training at early stage after stroke, similar gains were seen on the Fugl-Meyer and Berg balance scales in both groups [35, 48]. The results of this current study are consistent with published studies that early intervention can improve on balance and lower extremity motors functions in patients with subacute stroke. It is also consistent with Nilsson et al. [48] and Franceschini et al. [35] that BWSTT is at least as effective as CT to improve lower extremity functions and balance. The results indicate that two gait training strategies are similar in their clinical effects and could be used as routine therapeutic programs.

### 4.2. Kinematic Parameters

BWSTT is task-specific step training on treadmill with partial body weight support. Some research results showed that partial body supported on treadmill could result in better walking abilities than bearing their full weight of patients with stroke [31]. Other studies suggested that additional body weight support may reduce the stimulation to the affected side and may reduce the benefit in lower limb motor recovery [7]. Abnormal movement decreased after gait velocity improvement [49] and gait speed is a reliable outcome measure for short-term intervention in stroke patients. In this study, the subjects decreased body weight support from 40% at the beginning to zero by the end of the program. In addition, treadmill velocity was increased from 0.5 mph to 2.5 mph as early as possible, without causing abnormal changes in gait pattern to achieve optimal benefit of training [50, 51]. The results suggested that, after 3 weeks of BWSTT, improvement in temporal-spatial parameters (increased cadence, stride length and step length, and decreased stride time and step time) is achievable. Another research showed that a 12-week program of BWSTT coupled with overground walking practice led to increased floor walking speed with increased step lengths and cadence in patients with chronic stroke [52]. Result from this study indicated that patients with subacute stroke could improve gait velocity after just 3 weeks of BWSTT when combined with strength training, muscle recruitment facilitation, and occupational therapy programme. Improvement in gait speed is a result of increased stride length, step length, and cadence [53]. Previous study also demonstrated that BWSTT can improve gait velocity, cadence, and stride length parameters in subjects with subacute and chronic stroke [53]. BWSTT programme is superior to conventional therapy because it permits greater number of steps performed within a fixed period of time when compared to CT, thus increasing the amount of task-specific practice [32]. Hesse and Werner [54] reported up to 1000 steps which could be performed in a 20-minute treadmill training session, compared with only 50 to 100 steps during a 20-minute session of conventional physiotherapy. In addition, the speed of the treadmill, the amount of body weight support, and the amount of assistance provided by the physiotherapist can all be adjusted in order to provide a sufficient training intensity for individual needs. This can encourage further cortical reorganization and promote gait recovery.

It has been demonstrated that BWSTT improves symmetry and gait efficiency by changing kinematic parameters in acute stroke patients [32]. Increased paretic step length is due to an increased hip extension angle in the paretic leg that allows the limb to swing farther backward [51]. Hip extension increase was associated with meaningful changes in walking speed [55]. The kinematic findings illustrated that the paretic hip peak extension had increased significantly in BWSTT group but not in CT group and the increasing hip extension at terminal swing can contribute to rectify the increasing hip flexion at toe off with hemiparetic gait. This may explain the improvement in walking speed, stride length, step length, and cadence observed in BWSTT group but not in CT group. This is in agreement with study by Mulroy et al. [55] which also reported improvement in hip flexion and extension motion in stroke patient who has high response to gait training. Mulroy et al. [55] also reported a tendency toward greater increase in ankle plantarflexion motion, which is also in agreement with this study (see Figure 2). Study by Jonsdottir et al. [26] also indicated that the capacity to increase work production at the ankle may be limited. Faster treadmill walking speed could increase hip extension angle significantly and knee flexion during swing phase; the positive improvement facilitated a more normal gait pattern after stroke [56]. The exact reason why hip joint motions are more responsive than knee and ankle to gait training is currently unclear. Increase in hip extension/flexion angle requires increased joint power generation during walking. A possible explanation is that the hip joint consists of large muscle group such as rectus femoris and psoas major which are responsible for flexion and extension motion. It is possible that large muscles group that are responsible for mass motor movement are more responsive to intervention than smaller muscles group. Another possible explanation for the observed significant difference at the hip but not at ankle and knee is that the change of range of movement at the hip joint is the greatest among the three joints during a gait cycle. Therefore the sample size has sufficient power to detect significant difference at hip joint but not the other joints.

### 4.3. Relationship between Kinematic Parameters and Gait Pattern

The control of proximal and distal lower limb plays a key compensatory role in the adaptation of gait pattern and velocity in stroke patients in early stage [26, 27]. Lower extremity gait kinematics and kinetics in the sagittal plane are commonly impaired after stroke. This study showed improvement of hip extension in the BWSTT group increasing (from flexion 2.19° to extension 7.38°) and flexion decreased (from 29.01° to 22.92°). The kinematic curves showed that the hip joint was extended instead of flexed during stance phase. Previous joint angle analysis studies indicated that hip joint motion is more affected than all other lower limbs' joint among stroke survivors in early stage [4]. Hip extension angle improved when patients walked at progressively faster treadmill speeds [55, 57] and the gait speed increase was strongly associated with hip extension and ankle plantarflexion [55, 57, 58]. The improved hip motion is translated from stance to swing phase and enables effective ground reaction force to be generated anteriorly [59, 60]. It is therefore biomechanically important to promote hip joint movement to aid the forward propulsion of the body for stroke patient during gait [51].

It was found that lower extremity motor function and balance improved in both groups after 3 weeks of training. However, gait analysis revealed that gait speed and cadence improved in the BWSTT group, but not in the CT group over this time period. Furthermore, maximal hip extension improved with BWSTT and approached that in healthy controls, but there was no obvious difference in the CT group. The extent of hip extension may be related to the observed improvement in gait speed in this group. These results suggest that treadmill training with body weight support provided at an early stage after stroke may be beneficial to improve walking speed after stroke and abnormal flexion of hip joint at stance phase, especially in hyperflexion at hip joint during walking.

### 4.4. Kinetic Parameters

It is not known whether BWSTT training can target kinetic functions specially. Both BWSTT and CT groups in this study demonstrate some improvement in kinetic parameters after interventions but differences did not reach statistical significance. These findings would suggest that moment peak may not be related to the gait trajectories or gait speed improvement. Treadmill training is task specific to facilitate the development of new motor development after the new state following a stroke. It is possible that there was no direct relationship between improvement in muscle strength and gait improvement. However, patients with drop-foot have to increase the ankle joint plantarflexion moment during walking [61]. This study observed a decrease in plantarflexion moment after three weeks of intervention for ankle joint with BWSTT group. On the contrary, the plantarflexion moment increased in overground walking training. Therefore, it may be beneficial to adjust the abnormal gait curve. It is uncertain whether the lack of significant difference is due to the small sample size which lacks the power to detect small difference or the short three-week intervention period which was not sufficiently long to produce further changes.

## 5. Limitations

This study has several limitations. The sample size was small and was not power calculated to detect changes in gait parameter. This study only included stroke patients who were at the subacute stage. Results of this study may not be generalised to the larger stroke population. In addition, this study did not measure lower extremity muscle strength which limited the interpretation of the kinematic data. Thus, it is recommended that future research include muscle strength recording such as sEMG and isokinetic instruments, especially at hip flexor, knee extensor, and ankle plantarflexor, to understand the underlying mechanism.

## 6. Conclusion

BWSTT has similar clinical effects to improve balance and lower extremity function as conventional overground walking training for patients with subacute stroke. BWSTT can significantly improve spatiotemporal parameters with three weeks of training. Improvement in gait pattern is related to the improvement of hip joint motion during walking. It would be clinically beneficial to incorporate hip joint motion training to improve gait pattern, especially for those who are not suitable for treadmill training. Rehabilitation programme for subacute stroke patients should therefore incorporate kinematic training of proximal lower limb to facilitate gait recovery.

## Clinical Messages

- BWSTT is superior to conventional overground therapy in improving spatiotemporal parameters.
- Improvement in gait pattern is related to the improvement of kinematic pattern of proximal lower limb.
- Kinematic training of proximal lower limb may facilitate gait recovery.

## Figures and Tables

**Table 1:** Summary of demographic data.

Demographics	Normal group *X* (SD)	BWSTT group *X* (SD)	CT group *X* (SD)	*P*
Sex, number (%) of women	10 (83%)	10 (83%)	9 (75%)	
Affected side, left, number (%)	—	6 (50%)	6 (50%)	
Cerebral infarction, number (%)	—	11 (91.7%)	10 (83.3%)	
Age (y)	58.83 (8.03)	59.55 (9.23)	60.82 (10.70)	0.906
Body weight (kg)	63.08 (11.31)	65.17 (10.26)	65.25 (11.42)	0.987
Leg length (mm)	828.33 (42.12)	826.7 (53.78)	817.10 (60.1)	0.680
Days of postinjury	—	49.25 (19.51)	47.67 (16.78)	0.860

**Table 2:** Lower extremity function and balance score for normal group, BWSTT group, and CT group.

	Normal group *X* (SD)	BWSTT group *X* (SD)	CT group *X* (SD)	Between-group difference
MMSE score (0–30)	30	28.41 (1.16)	28.33 (1.37)	0.874
FMA (pre)	34	22.42 (4.36)	22.08 (6.44)	0.868
FMA (post)	—	24.33 (4.58)^*∗*^	25.17 (5.62)^*∗*^	0.633
Brunel (pre)	12	10.33 (1.30)	10.42 (1.38)	0.884
Brunel (post)	—	10.83 (1.44)^*∗*^	10.92 (1.68)^*∗*^	0.870

^*∗*^Differences between the period before and that after training were statistically significant.

**Table 3:** Spatiotemporal parameters for normal, BWSTT, and CT group.

Spatiotemporal parameters	Normal group *X* (SD)	BWSTT group *X* (SD) Before training	*X* (SD) After training	*P*	CT group *X* (SD) Before training	*X* (SD) After training	*P*
Cadence (steps/min)	106.75 (8.68)	64.59 (18.31)	85.41 (12.53)	0.005	70.10 (18.92)	70.34 (15.45)	0.953
Stride length (metres)	1.07 (0.10)	0.58 (0.16)	0.69 (0.18)	0.005	0.56 (0.10)	0.56 (0.15)	0.963
Stride time (seconds)	1.13 (0.09)	2.02 (0.64)	1.44 (0.21)	0.018	1.88 (0.67)	1.85 (0.69)	0.915
Step length (metres)	0.55 (0.06)	0.30 (0.07)	0.37 (0.09)	0.005	0.30 (0.07)	0.30 (0.08)	0.808
Step time (seconds)	0.57 (0.05)	1.06 (0.30)	0.81 (0.13)	0.015	1.08 (0.53)	1.00 (0.40)	0.655
Gait speed (m/s)	0.96 (0.10)	0.33 (0.17)	0.50 (0.20)	0.000	0.33 (0.12)	0.33 (0.12)	0.997

**Table 4:** Angle and moment peak of lower limb joints at sagittal plane.

	Normal group *X* (SD)	BWSTT group			CT group		
		*X* (SD)		*P*	*X* (SD)		*P*
		Before training	After training		Before training	After training	
*Kinematic parameters* (°)							
Hip flexion	28.69 (9.51)	29.01 (13.61)	22.92 (10.76)	0.021	25.03 (6.45)	22.75 (6.94)	0.308
Knee flexion	58.82 (10.25)	36.50 (10.15)	37.96 (15.95)	0.787	43.10 (11.71)	41.62 (13.71)	0.329
Ankle dorsiflexion	10.03 (7.76)	16.51 (17.02)	9.79 (5.42)	0.301	13.56 (6.03)	14.67 (4.16)	0.524
Hip extension	−11.78 (8.51)	1.31 (11.84)	−8.67 (10.78)	0.020	1.45 (6.62)	−0.75 (7.54)	0.453
Knee extension	4.41 (5.08)	2.49 (8.54)	−0.39 (8.51)	0.235	9.82 (7.01)	7.22 (6.09)	0.239
Ankle plantarflexion	−18.73 (7.76)	−11.56 (4.41)	−10.88 (11.53)	0.878	−6.72 (7.15)	−4.25 (6.95)	0.189

*Kinetic parameters* (Nm/kg)							
Hip flexion	0.72 (0.32)	0.36 (0.29)	0.50 (0.30)	0.061	0.17 (0.11)	0.20 (0.15)	0.702
Knee flexion	0.52 (0.39)	0.77 (0.50)	0.62 (0.47)	0.649	0.60 (0.42)	0.86 (0.51)	0.164
Ankle dorsiflexion	1.23 (0.32)	0.68 (0.40)	0.83 (0.35)	0.339	0.37 (0.38)	0.72 (0.47)	0.080
Hip extension	0.68 (0.31)	0.50 (0.28)	0.57 (0.38)	0.636	0.42 (0.15)	0.46 (0.28)	0.682
Knee extension	0.35 (0.09)	0.40 (0.33)	0.47 (0.37)	0.302	0.16 (0.14)	0.19 (0.14)	0.753
Ankle plantarflexion	0.15 (0.11)	0.76 (0.47)	0.51 (0.38)	0.099	0.56 (0.46)	0.67 (0.45)	0.541

## References

[1] Feigin V. L., Forouzanfar M. H., Krishnamurthi R. (2014). Global and regional burden of stroke during 1990–2010: findings from the Global Burden of Disease Study 2010. *The Lancet*.

[2] Zhao D., Liu J., Wang W. (2008). Epidemiological transition of stroke in China: twenty-oneyear observational study from the sino-MONICA-Beijing project. *Stroke*.

[3] Truelsen T., Bonita R. (2008). Epidemiological transition of stroke in China?. *Stroke*.

[4] McGinn A. P., Kaplan R. C., Verghese J. (2008). Walking speed and risk of incident ischemic stroke among postmenopausal women. *Stroke*.

[5] Collen F. M., Wade D. T., Bradshaw C. M. (1990). Mobility after stroke: reliability of measures of impairment and disability. *International Disability Studies*.

[6] Bohannon R. W., Andrews A. W., Glenney S. S. (2013). Minimal clinically important difference for comfortable speed as a measure of gait performance in patients undergoing inpatient rehabilitation after stroke. *Journal of Physical Therapy Science*.

[7] Hesse S. (2008). Treadmill training with partial body weight support after stroke: a review. *NeuroRehabilitation*.

[8] Hesse S. A., Jahnke M. T., Bertelt C. M., Schreiner C., Lücke D., Mauritz K.-H. (1994). Gait outcome in ambulatory hemiparetic patients after a 4-week comprehensive rehabilitation program and prognostic factors. *Stroke*.

[9] Perry S. B. (2004). Stroke rehabilitation: guidelines for exercise and training to optimize motor skill. *Journal of Neurologic Physical Therapy*.

[10] Nielsen R. K., Samson K. L., Simonsen D., Jensen W. (2013). Effect of early and late rehabilitation onset in a chronic rat model of ischemic stroke-assessment of motor cortex signaling and gait functionality over time. *IEEE Transactions on Neural Systems and Rehabilitation Engineering*.

[11] Liu X., Zhang S., Liu M. (2013). Chinese guidelines for endovascular management of ischemic cerebrovascular diseases. *Interventional Neurology*.

[12] Jiang B., Wang W.-Z., Chen H. (2006). Incidence and trends of stroke and its subtypes in China: results from three large cities. *Stroke*.

[13] Tilson J. K., Sullivan K. J., Cen S. Y. (2010). Meaningful gait speed improvement during the first 60 days poststroke: minimal clinically important difference. *Physical Therapy*.

[14] Drew T., Marigold D. S. (2015). Taking the next step: cortical contributions to the control of locomotion. *Current Opinion in Neurobiology*.

[15] Huppert T., Schmidt B., Beluk N., Furman J., Sparto P. (2013). Measurement of brain activation during an upright stepping reaction task using functional near-infrared spectroscopy. *Human Brain Mapping*.

[16] Olney S. J., Richards C. (1996). Hemiparetic gait following stroke. Part I: characteristics. *Gait & Posture*.

[17] Calautti C., Baron J.-C. (2003). Functional neuroimaging studies of motor recovery after stroke in adults: a review. *Stroke*.

[18] Yen C.-L., Wang R.-Y., Liao K.-K., Huang C.-C., Yang Y.-R. (2008). Gait training-induced change in corticomotor excitability in patients with chronic stroke. *Neurorehabilitation and Neural Repair*.

[19] Jette D. U., Latham N. K., Smout R. J., Gassaway J., Slavin M. D., Horn S. D. (2005). Physical therapy interventions for patients with stroke in inpatient rehabilitation facilities. *Physical Therapy*.

[20] Woldag H., Hummelsheim H. (2002). Evidence-based physiotherapeutic concepts for improving arm and hand function in stroke patients: a review. *Journal of Neurology*.

[21] Sawaki L., Wu C. W.-H., Kaelin-Lang A., Cohen L. G. (2006). Effects of somatosensory stimulation on use-dependent plasticity in chronic stroke. *Stroke*.

[22] Schaechter J. D., Van Oers C. A. M. M., Groisser B. N. (2012). Increase in sensorimotor cortex response to somatosensory stimulation over subacute poststroke period correlates with motor recovery in hemiparetic patients. *Neurorehabilitation and Neural Repair*.

[23] Celnik P., Hummel F., Harris-Love M., Wolk R., Cohen L. G. (2007). Somatosensory stimulation enhances the effects of training functional hand tasks in patients with chronic stroke. *Archives of Physical Medicine and Rehabilitation*.

[24] Miyai I., Yagura H., Oda I. (2002). Premotor cortex is involved in restoration of gait in stroke. *Annals of Neurology*.

[25] Luft A. R., Forrester L., Macko R. F. (2005). Brain activation of lower extremity movement in chronically impaired stroke survivors. *NeuroImage*.

[26] Jonsdottir J., Recalcati M., Rabuffetti M., Casiraghi A., Boccardi S., Ferrarin M. (2009). Functional resources to increase gait speed in people with stroke: strategies adopted compared to healthy controls. *Gait and Posture*.

[27] Chen C.-L., Chen H.-C., Tang S. F.-T., Wu C.-Y., Cheng P.-T., Hong W.-H. (2003). Gait performance with compensatory adaptations in stroke patients with different degrees of motor recovery. *American Journal of Physical Medicine and Rehabilitation*.

[28] Kim C. M., Eng J. J. (2004). Magnitude and pattern of 3D kinematic and kinetic gait profiles in persons with stroke: relationship to walking speed. *Gait and Posture*.

[29] Hesse S., Konrad M., Uhlenbrock D. (1999). Treadmill walking with partial body weight support versus floor walking in hemiparetic subjects. *Archives of Physical Medicine and Rehabilitation*.

[30] McCain K. J., Pollo F. E., Baum B. S., Coleman S. C., Baker S., Smith P. S. (2008). Locomotor treadmill training with partial body-weight support before overground gait in adults with acute stroke: a pilot study. *Archives of Physical Medicine and Rehabilitation*.

[31] Visintin M., Barbeau H., Korner-Bitensky N., Mayo N. E. (1998). A new approach to retrain gait in stroke patients through body weight support and treadmill stimulation. *Stroke*.

[32] Mehrholz J., Pohl M., Elsner B. (2014). Treadmill training and body weight support for walking after stroke. *The Cochrane Database of Systematic Reviews*.

[33] Mudge S., Rochester L., Recordon A. (2003). The effect of treadmill training on gait, balance and trunk control in a hemiplegic subject: a single system design. *Disability and Rehabilitation*.

[34] Werner C., Bardeleben A., Mauritz K.-H., Kirker S., Hesse S. (2002). Treadmill training with partial body weight support and physiotherapy in stroke patients: a preliminary comparison. *European Journal of Neurology*.

[35] Franceschini M., Carda S., Agosti M., Antenucci R., Malgrati D., Cisari C. (2009). Walking after stroke: what does treadmill training with body weight support add to overground gait training in patients early after stroke?: a single-blind, randomized, controlled trial. *Stroke*.

[36] da Cunha I. T., Lim P. A., Qureshy H., Henson H., Monga T., Protas E. J. (2002). Gait outcomes after acute stroke rehabilitation with supported treadmill ambulation training: a randomized controlled pilot study. *Archives of Physical Medicine and Rehabilitation*.

[37] MacKay-Lyons M., McDonald A., Matheson J., Eskes G., Klus M.-A. (2013). Dual effects of body-weight supported treadmill training on cardiovascular fitness and walking ability early after stroke: a randomized controlled trial. *Neurorehabilitation and Neural Repair*.

[38] Moreno J. C., Barroso F., Farina D. (2013). Effects of robotic guidance on the coordination of locomotion. *Journal of NeuroEngineering and Rehabilitation*.

[39] Routson R. L., Clark D. J., Bowden M. G., Kautz S. A., Neptune R. R. (2013). The influence of locomotor rehabilitation on module quality and post-stroke hemiparetic walking performance. *Gait and Posture*.

[40] Fugl Meyer A. R., Jaasko L., Leyman I. (1975). The post stroke hemiplegic patient. I. A method for evaluation of physical performance. *Scandinavian Journal of Rehabilitation Medicine*.

[41] Tyson S. F., DeSouza L. H. (2004). Development of the Brunel Balance Assessment: a new measure of balance disability post stroke. *Clinical Rehabilitation*.

[42] Tyson S. F., Hanley M., Chillala J., Selley A. B., Tallis R. C. (2007). The relationship between balance, disability, and recovery after stroke: predictive validity of the Brunel Balance Assessment. *Neurorehabilitation and Neural Repair*.

[43] Sheffler L. R., Bailey S. N., Wilson R. D., Chae J. (2013). Spatiotemporal, kinematic, and kinetic effects of a peroneal nerve stimulator versus an ankle foot orthosis in hemiparetic gait. *Neurorehabilitation and Neural Repair*.

[44] Jones T. A., Kleim J. A., Greenough W. T. (1996). Synaptogenesis and dendritic growth in the cortex opposite unilateral sensorimotor cortex damage in adult rats: a quantitative electron microscopic examination. *Brain Research*.

[45] Myint J. M. W. W., Yuen G. F. C., Yu T. K. K. (2008). A study of constraint-induced movement therapy in subacute stroke patients in Hong Kong. *Clinical Rehabilitation*.

[46] Katz-Leurer M., Sender I., Keren O., Dvir Z. (2006). The influence of early cycling training on balance in stroke patients at the subacute stage. Results of a preliminary trial. *Clinical Rehabilitation*.

[47] Richards C. L., Malouin F., Wood-Dauphinee S., Williams J. I., Bouchard J.-P., Brunet D. (1993). Task-specific physical therapy for optimization of gait recovery in acute stroke patients. *Archives of Physical Medicine and Rehabilitation*.

[48] Nilsson L., Carlsson J., Danielsson A. (2001). Walking training of patients with hemiparesis at an early stage after stroke: A comparison of walking training on a treadmill with body weight support and walking training on the ground. *Clinical Rehabilitation*.

[49] Kramers De Quervain I. A., Simon S. R., Leurgans S., Pease W. S., McAllister D. (1996). Gait pattern in the early recovery period after stroke. *The Journal of Bone and Joint Surgery—American Volume*.

[50] Aaslund M. K., Helbostad J. L., Moe-Nilssen R. (2013). Walking during body-weight-supported treadmill training and acute responses to varying walking speed and body-weight support in ambulatory patients post-stroke. *Physiotherapy Theory and Practice*.

[51] Tyrell C. M., Roos M. A., Rudolph K. S., Reisman D. S. (2011). Influence of systematic increases in treadmill walking speed on gait kinematics after stroke. *Physical Therapy*.

[52] Plummer P., Behrman A. L., Duncan P. W. (2007). Effects of stroke severity and training duration on locomotor recovery after stroke: a Pilot Study. *Neurorehabilitation and Neural Repair*.

[53] Ada L., Dean C. M., Hall J. M., Bampton J., Crompton S. (2003). A treadmill and overground walking program improves walking in persons residing in the community after stroke: a placebo-controlled, randomized trial. *Archives of Physical Medicine and Rehabilitation*.

[54] Hesse S., Werner C. (2003). Poststroke motor dysfunction and spasticity: novel pharmacological and physical treatment strategies. *CNS Drugs*.

[55] Mulroy S. J., Klassen T., Gronley J. K., Eberly V. J., Brown D. A., Sullivan K. J. (2010). Gait parameters associated with responsiveness to treadmill training with body-weight support after stroke: an exploratory study. *Physical Therapy*.

[56] Krawczyk M., Szczerbik E., Syczewska M. (2014). The comparison of two physiotherapeutic approaches for gait improvement in sub-acute stroke patients. *Acta of Bioengineering and Biomechanics*.

[57] Hesse S., Uhlenbrock D., Sarkodie-Gyan T. (1999). Gait pattern of severely disabled hemiparetic subjects on a new controlled gait trainer as compared to assisted treadmill walking with partial body weight support. *Clinical Rehabilitation*.

[58] Hsu A.-L., Tang P.-F., Jan M.-H. (2003). Analysis of impairments influencing gait velocity and asymmetry of hemiplegic patients after mild to moderate stroke. *Archives of Physical Medicine and Rehabilitation*.

[59] Turns L. J., Neptune R. R., Kautz S. A. (2007). Relationships between muscle activity and anteroposterior ground reaction forces in hemiparetic walking. *Archives of Physical Medicine and Rehabilitation*.

[60] Bowden M. G., Balasubramanian C. K., Neptune R. R., Kautz S. A. (2006). Anterior-posterior ground reaction forces as a measure of paretic leg contribution in hemiparetic walking. *Stroke*.

[61] Simonsen E. B. (2014). Contributions to the understanding of gait control. *Danish Medical Journal*.

